# HLA class II and rheumatoid arthritis: the bumpy road of revelation

**DOI:** 10.1007/s00251-017-0987-5

**Published:** 2017-07-11

**Authors:** Arieke S.B. Kampstra, René E.M. Toes

**Affiliations:** Department of Rheumatology, Leiden University Medical Center, PO Box 9600, 2300RC Leiden, The Netherlands

**Keywords:** Rheumatoid arthritis, HLA class II molecules, T cells, ACPA+ disease, Shared epitope, DERAA

## Abstract

Rheumatoid arthritis (RA) is a chronic auto-immune disease primarily targeting the joints. Approximately 1% of the population is affected by RA, and despite the improvements in therapeutic interventions, elucidation of the disease pathogenesis is still in its infancy. RA patients can be subdivided on basis of the presence of autoantibodies, especially anti-citrullinated protein antibodies (ACPA). ACPA^+^ and ACPA^−^ disease most likely differ in aetiology, as different genetic and environmental risk factors are associated with these two disease entities. For ACPA^+^ RA disease, the genetic factors associating with disease mainly comprised of human leukocyte antigen (HLA) class II molecules. The predisposing HLA-DR alleles have been depicted as the ‘HLA Shared Epitope (SE) alleles’, as these alleles encode a similar sequence, the shared epitope sequence, within the beta chain of the HLA-DR molecule. In addition to the involvement of the HLA-SE alleles in the development of ACPA^+^ RA disease, other HLA-DR molecules have been shown to confer protection against this disease entity. The protective HLA molecules have, instead of the SE-motif, a different but shared sequence at the same location in the beta chain of HLA-DR molecules, consisting of the amino acid residues DERAA. The possible contributions of the predisposing and protective HLA molecules in association with ACPA-positive RA are discussed in this review.

## Introduction

Rheumatoid arthritis (RA) is a chronic auto-inflammatory disease that primarily affects the joints. Worldwide the prevalence ranges between 0.5 and 1% with a few populational exceptions and a higher occurrence in females than in males (Silman & Pearson, 2002). One of the first humoral markers to be identified within the RA patient population has been the presence of rheumatoid factor (RF), an antibody that targets the Fc region of immunoglobulin G (IgG) molecules (Zvaifler, 1973). However, this specific antibody is also present in a variety of other diseases. Another antibody response with higher specificity for RA was shown to target citrullinated proteins. Citrullination is a posttranslational modification converting peptidylarginine residues into peptidylcitrulline involving the peptidylarginine deiminase (PAD) enzyme. A consequence of this modification is the reduction of the net-positive charge of the protein. Not all RA patients produce these antibodies, making a distinction between the RA populations that do- or do not harbour Anti-citrullinated protein antibodies (ACPA). ACPA positivity has been shown to be a predictive marker for the development of RA in patients with joint complaints (van Gaalen et al., 2004) and patients harbouring ACPAs experience a more aggressive disease progression than patients without ACPA (van der Helm-van Mil, Verpoort, Breedveld, Toes, & Huizinga, 2005). This distinction between these two disease entities was further emphasized by differences in underlying genetic risk factors and environmental risk factors, pointing towards a different aetiology and pathophysiology between ACPA+ and ACPA− RA disease.

### HLA associations with RA

In several twin studies, the heritability of RA was estimated to be approximately 60%, pointing towards a substantial influence of genetic risk factors on the development of RA disease (MacGregor et al., 2000). Recent genome-wide association studies (GWAS) have identified 101 single nucleotide polymorphism (SNPs) in total, showing the highest contribution of the *hla-drb1* gene to the development of RA (Eyre et al., 2012; Okada et al., 2012; Okada et al., 2014b; Stahl et al. 2010). *Hla-drb1-*encoded proteins are components of human leukocyte antigen-DR (HLA-DR) molecules and together with HLA-DQ and HLA-DP, they represent the major determinants in the induction of adaptive immune responses. They are expressed, amongst others, by antigen-presenting cells (APCs) and are able to present peptides to CD4^+^ T cells. In the 1970s, HLA-Dw4 was shown to be present in the majority of the RA patients (Stastny 1976) which was confirmed by serological HLA-typing identifying HLA-DR4 and HLA-DR1 in association with RA. Nowadays, the list of HLA alleles conferring increased risk for RA development is largely known (listed in Table 1) albeit with altered nomenclature. The predisposing HLA-DR alleles were found to have a particular sequence in common, located in the beta chain (HLA-DRB1) at positions 70–74 (Gregersen et al. 1987). This has later become known as the shared epitope sequence and as such, the HLA-DR alleles carrying this particular sequence were designated as ‘Shared Epitope alleles’(SE-alleles). In 2005, it was discovered that the genetic contribution of the HLA locus did not apply to RA as such, but rather to ACPA-positive RA only (Huizinga et al. 2005). These data are important as they indicate that ACPA-positive and ACPA-negative RA represent different disease entities with a different underlying pathophysiology. More recently, positions 11 and 13 (Table 1), which are also part of the peptide-binding groove, have been implicated in the association between HLA and RA (Raychaudhuri et al. 2012). However, as these positions are the most polymorphic in the HLA-region, these two positions most likely represent the best proxy for the predisposing HLA molecules explaining their association with RA in statistical terms.

**Table 1:**
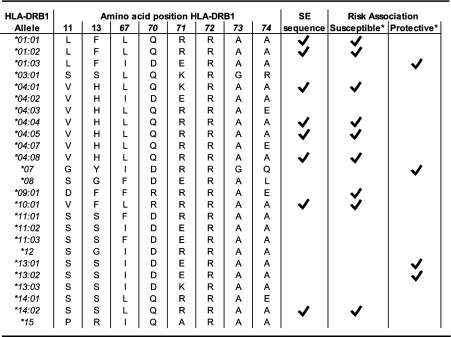
Association of HLA-DRB1 alleles with ACPA^+^ RA disease. Depicted are the residues located at various positions for a diversity of HLA-DRB1 alleles. Not all known HLA alleles are shown here. The presence of the SE-sequence is indicated with a tick, as well as the association with predisposition or protection against ACPA^+^ RA disease for each allele

### Shared epitope alleles and RA

#### Epidemiology

The contribution of HLA alleles to RA development has been extensively studied by means of meta-analyses in different populations, including the Asians, Caucasians and native Americans. These studies show that especially the HLA-SE alleles increase the risk of developing RA in every population, although there are discrepancies in the degree of contribution of each allele when comparing populations (Okada et al. 2014a; van der Woude et al. 2010a; Willkens et al. 1991). The presence of combined SE-alleles within individuals increases the risk of developing ACPA+ RA even more (Mackie et al. 2012). Interestingly, even though the SE-alleles confer the highest risk, some non-SE alleles have also been identified as predisposing. For example, HLA-DRB1*09:01 is not considered a shared epitope allele by a small difference in the five residues located at positions 70–74 (Bondinas et al. 2007); however, in meta-analyses, the presence of HLA-DRB1*09:01 has shown an increase in odds ratio (van der Woude et al. 2010a; Willkens et al. 1991). This notion indicates that the involvement of HLA class II molecules may be dependent on more than just the shared epitope sequence.

#### Citrulline presentation by shared epitope alleles

**Fig. 1:**
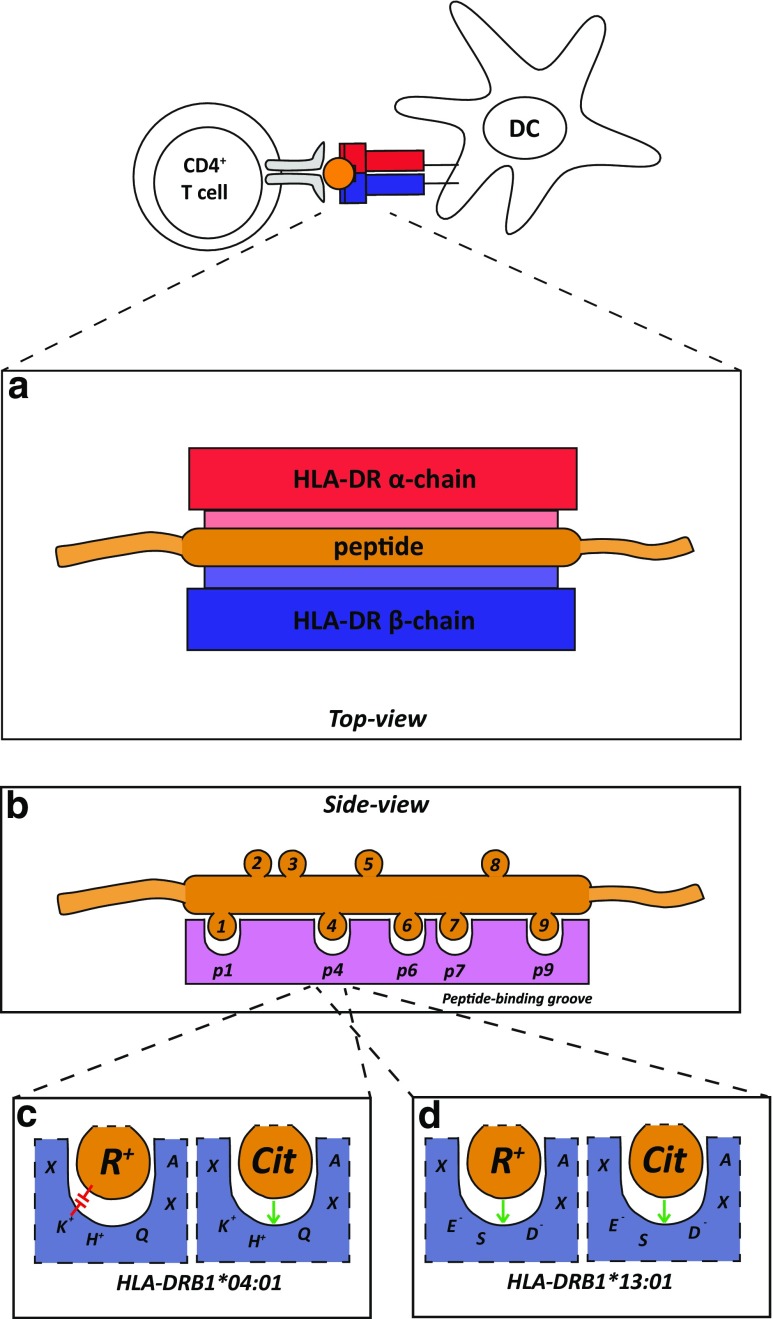
Schematic representation of a peptide binding to a HLA-DR molecule. **a** Top view of the peptide binding groove. The peptide binding groove is formed by the alpha and beta chain, leaving, forming two walls to accommodate the peptide. **b** Side-view of the peptide-binding groove without distinguishment between the two HLA-DR chains. The peptide-binding pockets shown are anchoring points for residues of the peptide. The other residues are available for T cell receptor recognition. **c** Representation of how the shared epitope sequence can influence the binding of residues. Due to the presence of positively charged residues within pocket 4, arginine may be repelled and citrulline may be accepted. X represents any amino acid involved in formation of pocket 4. Position of residues within peptide-binding pocket 4 does not represent the actual position. **d** Representation of how the DERAA-sequence can influence the binding of residues. Due to the presence of negatively charged residues within pocket 4, both arginine and citrulline may be accepted. X represents any amino acid involved in formation of pocket 4. Position of residues within peptide-binding pocket 4 does not represent the actual position

HLA class II molecules consist of 2 protein chains, the alpha and beta chain (Fig. 1a), both encoded by different genes. The combination of the two chains together forms a molecule with a peptide-binding groove for the presentation of peptides (Fig. 1a). This peptide-binding groove consists of 4–5 peptide-binding pockets, accommodating different residues of the bound peptide ligand (Fig. 1b). The amino acids involved in the formation of these pockets are key determinants in restricting the peptide repertoire that can be accommodated. Even though peptides of variable lengths can bind to HLA class II molecules, the core sequence of these peptides consists of 9 amino acids. Of these 9 amino acids, 4–5 residues will get enclosed by the HLA molecule, in peptide-binding pockets 1, 4, 6, (7) and 9, whereas the remainder is available for T cell receptor (TCR) recognition (Fig. 1b).

The previously mentioned shared epitope sequence either consists of the residues ^70^QKRAA^74^, ^70^QRRAA^74^ or ^70^RRRAA^74^. Three (residue 70, 71 and 74) out of the five amino acids are involved in the shaping of peptide-binding pocket 4 of the HLA molecule. The presence of one or two positively charged residues (lysine, K; arginine, R) within the pocket alters the permissiveness of the binding of positively-charged residues at that position, enabling enhanced binding capacity of citrullinated peptides compared to the native peptide (Fig. 1c). To confirm this enhanced binding capacity, Hill et al. performed peptide-binding assays showing that the HLA-SE molecules were capable of binding citrullinated peptides with higher affinity than their native counterparts (Hill et al. 2003). Indeed, subsequent HLA-DRB1*04:01-peptide crystal structures clearly showed that this HLA-molecule exhibited an enhanced ability to accommodate citrulline, but not arginine, within peptide-binding pocket 4 (Scally et al. 2013). However, recent evidence indicates that not only the Shared Epitope motif within peptide-binding pocket 4 contributes to citrulline binding, as pockets 7 and 9 are also capable of accommodating citrulline more efficiently. In addition, we have shown that HLA-DQ molecules, that are in tight linkage-disequilibrium (LD) with predisposing HLA-DR molecules (HLA-DQ8 and –DQ7, in LD with HLA-DR4), but also ‘neutral’ HLA-DQ molecules (HLA-DQ2, in LD with HLA-DR3), are able to accommodate citrullinated peptides better as their non-modified counterparts. Moreover, they can do so in multiple pockets (Kampstra et al. 2016). Therefore, the association between SE-alleles and the development of ACPA+ RA disease may not be explained fully by enhanced binding of citrullinated peptides by these alleles as the interactions between citrullinated peptides and HLA molecules seem to be more complex. In addition, due to the tight LD between HLA-DR and –DQ molecules, it is complicated to define the precise contribution of these molecules to RA pathogenesis.

#### T cell recognition of citrullinated peptides

In contrast to the increasing data on the ACPA response, including the ACPA-expressing B cell response, knowledge on the nature of the T cells involved in RA is limited. As mentioned previously, the HLA class II molecules present peptides to activate and/or modulate the CD4^+^ T cell response. T cells can exert a diversity of functions including the promotion of the immune response by helping B cells. Recent studies have shown that the HLA class II locus is associated with established ACPA^+^ disease, but, surprisingly, only to a limited extend with ACPA-positivity in healthy subjects. Therefore, it is likely that the CD4^+^ T helper cells restricted by the predisposing HLA molecules play a role in the development of ACPA^+^ disease rather than in the development of ACPA positivity. The contribution of this T cell-help may lie in the facilitation of the maturation of the antibody response by B cells, as relatively short before the onset of symptoms, the antibody response has been shown to expand in level, isotype usage and fine specificity recognition profile (Suwannalai et al. 2012; van de Stadt et al. 2011; van der Woude et al. 2010b; Verpoort et al. 2006). Despite the increasing knowledge on the nature of citrullinated antigens recognized by ACPAs, knowledge on T cell receptor specificity is still scarce. Next-generation sequencing has shown that there might be oligoclonal expansion of T cell populations; nevertheless, common TCR specificities were not identified among different RA patients (Klarenbeek et al. 2012). The difficulty in identifying the T cell reaction involved in RA pathogenesis might be partially due to the cross-reactive nature of ACPA, being able to recognize many citrullinated proteins. Therefore, the antigens recognized by B cells in vitro are not necessarily the same antigens that are recognized by T cells in vivo, making it challenging to pinpoint a specific dominant T cell response in RA patients. Moreover, because of the cross-reactive nature of ACPA, it is conceivable that the auto-reactive ACPA-expressing B cells also react to citrullinated proteins from microbes, thereby allowing the recruitment of microbe-directed T cells, instead of auto-reactive T cells, in the help provided to ACPA-producing B cells. Therefore, it has yet to be elucidated whether and to what extent T cells are auto-reactive in nature. Despite these challenges, studies have shown the existence of T cells specific for citrullinated peptides in mice. For example, HLA-DR4 (HLA-DRB1*04:01) transgenic mice immunized with 2 naturally processed peptides derived from citrullinated vimentin, readily respond to these with the production of interferon-gamma. This response was both restricted to the citrullinated form of the peptide and to the presentation of the peptide in the context of HLA-DR (Feitsma et al. 2010; James et al. 2014). In addition, multiple studies suggest that such citrullinated peptide-specific T cells can also be present in RA patients, although they are also found in healthy controls (Hill et al. 2003; James et al. 2014; Scally et al. 2013).

### HLA-DR13 and RA

#### Epidemiology

Since the identification of the HLA-SE alleles, the majority of studies have focused on the association between the presence of HLA-SE alleles and RA. Another facet of the HLA association with ACPA^+^ RA is found in the presence of protective HLA alleles. These protective HLA alleles have been categorized according to three classifications: the presence of the sequence ‘DERAA’ at positions 70–74 of the beta chain, the presence of an aspartic acid at position 70 or the presence of an isoleucine at position 67 (Table 1). An extensive meta-analysis on the influence of multiple HLA alleles has shown that despite these categories, HLA-DRB1*13:01, and to a lesser extent HLA-DRB1*13:02, confers protection in ACPA^+^ RA but not ACPA^−^ RA in four different populations (Salvat et al. 1994; van der Woude et al. 2010a). In line with these observations, a different study has shown that HLA-DRB1*13 confers protection against ACPA^+^ RA rather than ACPA positivity (van Heemst et al. 2016). The mechanism how HLA molecules can protect against ACPA^+^ disease development is yet to be elucidated; nevertheless, several ideas have been postulated.

#### Citrulline presentation by protective HLA molecules

Despite the in-depth analyses of the association between HLA-DRB1*13 and ACPA+ RA disease, only limited data are available on the ability of HLA-DRB1*13, or other protection-associated HLA molecules, to bind citrullinated or native peptides. Nonetheless, in contrast to the predisposing HLA molecules, the HLA molecules associated with protection seem to be able to accommodate both citrullinated- and native peptides with similar efficiency as reported using a model peptide from vimentin (Hill et al. 2003; Scally et al. 2013) (Fig. 1d). Therefore, it was proposed that due to the increased binding affinity of citrulline for the predisposing HLA molecules, the number of peptide-HLA complexes is increased on the cell surface as opposed to the neutral or protective peptide-HLA complexes (Hill et al. 2003). As the level of peptides presented by protective HLA molecules is less likely to reach the threshold for T cell activation, the so-called ‘biochemical margin of safety’ (Hill et al. 2003; Peterson et al. 1999), it will not lead to T cell activation or might even lead to induction of regulatory T cells (Jordan et al. 2001; Peterson et al. 1999). In addition, peptides that bind with higher affinity to HLA molecules increase the lifespan of the peptide-HLA complex on the cell surface, increasing the opportunity of T cells to recognize the presented peptide (Nelson et al. 1994). However, whether these features are related to the immunogenetic data remains a question that is yet to be elucidated.

#### HLA-DERAA presentation by thymic APCs

HLA molecules can present an array of peptides derived from endogenous proteins whereby the peptides presented by HLA class II molecules are often derived from proteins from the cell membrane (Rammensee and Friede, 1995). Therefore, it is no surprise that HLA class II molecules also present peptide derived from other HLA proteins (Chicz et al. 1992). Given the protective effects associated with some HLA molecules, it has been proposed that the presentation of a specific epitope derived from HLA molecules protect against development of arthritis through the induction of regulatory T cells recognizing such peptides. For example, it was proposed that the murine MHC molecule H-2Eb^d^ is able to confer protection through the presentation of a peptide derived from its high variable domain 3 (HV3) by H-2A^q^ (Zanelli et al. 1995). In this case, it was hypothesised that protection would be mediated through the induction of anergic, regulatory T cells. Because presentation of peptides derived from MHC class II molecules by other MHC class II molecules could potentially lead to the formation of regulatory T cell responses, it was proposed that T cells specific for the HV3 region are positively selected in the thymus and subsequently could contribute to immune regulation and inhibition of auto-immunity in mice and men (Zanelli et al. 1995). Indeed, it has been shown that a peptide derived from the protective HLA-DRB1*04:02, but not from HLA-DRB1*0401, can be presented by HLA-DQ8 and be recognized by HLA-DQ8-restricted T cells, supporting the notion that peptides from the HV3 region of HLA molecules can serve as T cell targets (Snijders et al. 2001).

#### HLA-DR13 and RA: how could they connect?

A possible biological explanation of the protective effects associated with HLA-DR13 was found in the presence of the sequence ^70^DERAA^74^ encoded by HLA-DR13 and other protective HLA molecules (van Heemst et al. 2015). Next to several HLA alleles associated with protection against ACPA^+^ RA, the DERAA-sequence can also be found in a few other human self-proteins including vinculin (VCL). Vinculin is a component of the cytoskeleton, ubiquitously expressed in all cell lineages. It has been shown to be citrullinated within the inflamed joints of RA patients (van Beers et al. 2013), and to be recognized by ACPA in its citrullinated form (van Heemst et al. 2015). Moreover, T cell tolerance against vinculin had previously been reported not to be absolute (Propato et al. 2001) as under certain pathological conditions the presence of T cells recognizing vinculin have been reported. Interestingly, comparing HLA-DRB1*13:01-positive and HLA-DRB1*13:01-negative individuals revealed that T cell reactivity against ‘vinculin-DERAA’ was readily detectable in many HLA-DR13-negative individuals, but absent in HLA-DR13-positive subjects. Moreover, when binding affinity of the ‘VCL-DERAA’ peptide to different HLA molecules was examined, it was shown that the peptide was able to bind to all HLA-DQ molecules that are associated with ACPA-positive RA (i.e. HLA-DQ7, -DQ8 and -DQ5) as these molecules are in strong linkage disequilibrium with predisposing HLA-DR molecules. In contrast, the HLA molecules analysed that do not associate with arthritis, did not bind this peptide, suggesting a link between the ability to present this peptide and the predisposition to ACPA^+^ RA. Interestingly, it was subsequently shown that a T clone against the ‘VCL-DERAA’ peptide also cross-reacted to several ‘DERAA’-containing epitopes present in microbes. These peptides bound the predisposing HLA-DQ molecules as well and were recognized by a substantial number of HLA-DR4-DQ8/7.3-positive donors. However, such T cells remained undetectable in donors additionally expressing the protective HLA-DR13 molecule. Therefore, a possible mechanism explaining the presence of ‘VCL-DERAA’-specific T cells is found in the notion that a breach of tolerance is induced following exposure to a microbe-harbouring protein that resembles the VCL-DERAA sequence. This could lead to the induction of cross-reactive T cells that recognize the auto-antigen vinculin. Intriguingly, the DERAA-sequence is present in 66% of bacteria and 4% of viruses, indicating that everyone will be exposed to such microbes. Thus, taking both the ability of a VCL-DERAA-directed T cells to respond to ‘DERAA’-containing epitopes from microbes, and the apparent selective absence of reactivity of such T cells within HLA-DR13^+^ individuals, an hypothesis describing the connection between HLA and RA has been proposed. In short, upon encounter and peripheral priming by microbial-derived DERAA-containing peptides, ‘DERAA’-directed T cells will be activated in individuals expressing HLA molecules that can present such peptides (i.e. the HLA-DQ molecules predisposing to RA). Some of these T cells show cross-reactivity with VCL-DERAA and thereby allow the provision of help to ACPA-expressing B cells that recognize citrullinated vinculin (a target of ACPA that is also present in citrullinated form in the inflamed joint). In contrast, individuals carrying the protective HLA molecules will not be able to mount such T cell responses as these T cells are hypothesised to be negatively selected in the thymus by presentation of the DERAA-sequence derived from protective HLA molecules. Therefore, by virtue of the absence of such T cells, ACPA-expressing B cells recognizing citrullinated vinculin will not receive T cell-help explaining the protective effects of HLA-DR13 to ACPA-positive RA (van Heemst et al. 2015).

## Concluding remarks

The association between the HLA-system and RA has been studied intensively in the past 40 years thereby addressing many aspects involved in the pathogenesis of RA. Even though many breakthroughs have been made, there is yet so much more to be elucidated, primarily within the context of which role T cells play during the development of ACPA^+^ RA. The elucidation of the exact events underlying the development of ACPA^+^ RA might provide relevant insights into the role of T cells and peptide presentation by HLA class II molecules and thereby the pathogenesis of this frequent auto-immune disease. It is likely that many aspects on the role of T cells in ACPA^+^ disease will be clarified in the years to come, most likely unveiling several pathways the T cells employ to contribute to the arousal of ACPA^+^ RA.
